# Prevalence of Mental Health Discussions in Publicly Available Generative AI Conversations

**DOI:** 10.1001/jamanetworkopen.2026.30635

**Published:** 2026-08-24

**Authors:** Ryan K. McBain, Li Ang Zhang, Alyssa Burnett, Jonathan H. Cantor, Hao Yu

**Affiliations:** 1 Health Care Division, RAND, Washington, DC; 2 Harvard Medical School, Boston, Massachusetts; 3 Health Care Division, RAND, Santa Monica, California; 4 Harvard Pilgrim Health Care Institute, Boston, Massachusetts

## Abstract

This cross-sectional study evaluates how often adolescents use artificial intelligence (AI) chatbots to discuss mental health concerns.

## Introduction

Artificial intelligence (AI) chatbots are increasingly used for social, relational, and emotional guidance, as they provide advice in situations ranging from everyday stress to explicit mental health crises.^1^ Technology companies, researchers, and news outlets have begun estimating how often these interactions occur, but their estimates diverge sharply. One AI developer has concluded that 0.01% of messages in a given week “indicate possible signs of mental health emergencies related to psychosis or mania,”^2^ while Common Sense Media has reported that 72% of teenagers have used AI chatbots as “companions,” a framing that implies a broad social-emotional role for AI in adolescents’ lives.^3^ These figures may not represent competing measurements of a single phenomenon; they may reflect different operational definitions of what counts as mental health use. We aimed to quantify how prevalence varies under conservative vs expansive definitions of mental health by using a large public dataset of chatbot transcripts.

## Methods

The study followed the STROBE reporting guideline for cross-sectional studies. Because the dataset was publicly available and deidentified, the study was deemed exempt from institutional review board oversight and informed consent by Harvard Pilgrim Health Care Institute.

We conducted a cross-sectional analysis of 620 699 ChatGPT (OpenAI) conversations from WildChat-4.8M, a publicly available corpus of human-chatbot conversations.^4^ The corpus includes conversation text and limited metadata. We developed a large language model (LLM)–based classifier to identify mental health–related conversations and assign ordinal scores (0, absent; 1, limited or peripheral presence; 2, central presence) across 4 domains: (1) topicality, defined as the centrality of mental health content; (2) intent, defined as the degree of help-seeking or guidance seeking; (3) clinical language, defined as use of diagnostic or treatment terminology; and (4) affective risk, defined as severity of distress or crisis. Operational definitions and scoring criteria were developed using pilot conversations and finalized before classifier validation.

Classifier performance was assessed in a randomly selected 370-conversation validation sample independently coded by 2 human reviewers (J.H.C. and A.B.) using the finalized criteria and a binary label for whether the conversation was mental health related. Discordant ratings (22 of 370 [6%]) were adjudicated by a third human reviewer (R.K.M.) blinded to initial reviewers’ ratings. Human reviewers’ raw agreement was 94%, with interrater reliability of κ = 0.65. Classifier performance was summarized using sensitivity, specificity, positive predictive value (PPV), and negative predictive value (NPV). Additional details on the LLM classifier, including the model, manufacturer, and version are provided in the eMethods in Supplement 1.

We applied the LLM classifier to the analytic dataset to calculate prevalence estimates, with 95% CIs estimated using binomial methods to reflect sampling uncertainty. Estimates quantified 2 constructs: (1) a conservative definition requiring an identifiable real person as the participant and explicit help-seeking for a psychological problem and (2) an expansive definition requiring a topicality score of 1 or greater, regardless of identifiable person focus or explicit help-seeking. Total scores across domains were quantified descriptively, not as classification cutoffs. Statistical analysis was conducted with R version 4.6.1 (R Project for Statistical Computing).

## Results

Against adjudicated human ratings as the reference standard, the LLM-based classifier demonstrated sensitivity of 87%, specificity of 95%, PPV of 67%, and NPV of 98%. Under the conservative definition of what counts as a mental health conversation, 1317 of the 620 699 conversations were classified as mental health related (0.21%; 95% CI, 0.20%-0.22%). Under the expansive definition, 30 394 of 620 699 conversations were classified as mental health related (4.90%; 95% CI, 4.84%-4.95%), a 23-fold difference (Figure).

**Figure.**
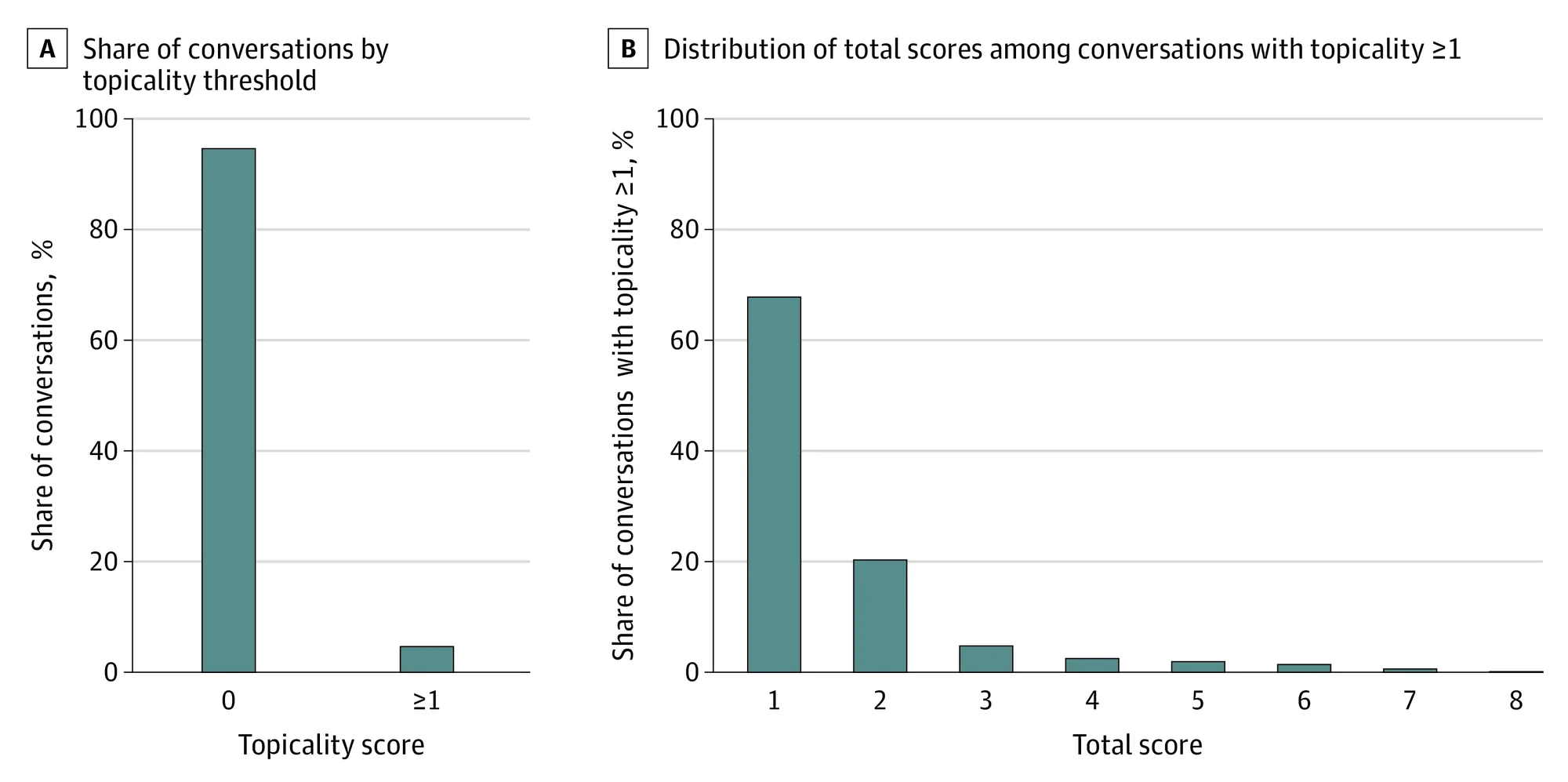
Bar Graphs of Conversations by Topicality and Total Score

Conversations captured by the conservative definition had higher topicality and intent scores, consistent with the definition’s requirement for explicit help-seeking. Conversations captured by the expansive definition more often met criteria for mental health topicality without meeting criteria for identifiable person focus or explicit help-seeking.

## Discussion

In a large public dataset of human-chatbot conversations, estimated mental health prevalence varied by more than an order of magnitude, depending on definitional scope. This finding helps reconcile why prevalence estimates in public discourse can range from rare crisis signals to widespread engagement. Definition-dependent reporting is especially consequential because duties of care and safety evaluation thresholds hinge on whether an interaction is recognized as mental health.^5,6^

We recommend future surveillance and evaluation of AI conversations report results using a tiered taxonomy that distinguishes: (1) crisis or high-risk conversations, (2) clinically framed conversations, and (3) broad affective or interpersonal conversations; such a taxonomy could have practical value for aggregate monitoring and auditing. Crisis or high-risk conversations require reliable detection and escalation safeguards; clinically framed help-seeking requires evaluation of accuracy, clinical appropriateness, and referral behavior; and broad affective or interpersonal conversations may require attention to emotional dependence and developmental appropriateness. Standardized reporting would help researchers, clinicians, and regulators benchmark chatbot performance, monitor changes as models and platform policies evolve, identify safety gaps, and determine where oversight is warranted.

Study limitations include reliance on a public corpus of transcripts without external validation. In addition, although the classifier was validated against adjudicated human ratings, automated labeling may misclassify ambiguous expressions of distress, and adjudication of discordant human ratings may introduce judgment in borderline cases. Additionally, this study did not evaluate whether taxonomy-based monitoring would improve population health outcomes.

## Notes

Supplement 1.
eMethods.

Supplement 2.
Data Sharing Statement
