# Supplementary material for: Prevalence of Mental Health Discussions in Publicly Available Generative AI Conversations
Source: JAMA Netw Open. 2026 Aug 24;9(8):e2630635. doi: 10.1001/jamanetworkopen.2026.30635 (PMC13504430; doi:10.1001/jamanetworkopen.2026.30635)
Supplement: Supplementary file 1 [file jamanetwopen-e2630635-s001.pdf]

# Supplemental Online Content

McBain RK, Zhang LA, Burnett A, Cantor JH, Yu H. Prevalence of mental health discussions in publicly available generative AI conversations. *JAMA Netw Open.* 2026;9(8):e2630635. doi:10.1001/jamanetworkopen.2026.30635

**eMethods.**

This supplemental material has been provided by the authors to give readers additional information about their work.

© 2026 McBain RK et al. *JAMA Network Open*.

**eMethods.**

1. **Use of Artificial Intelligence in Research**

We used a large language model (LLM) as an analytic classifier to identify mental health-related conversations and assign scores across four domains: topicality, intent, clinical language, and affective risk. The classifier used Grok 3 (grok-3-v1), developed by xAI, accessed through Azure AI Foundry between November 2025 to December 2025. The LLM was used only for classification of deidentified, publicly available conversations and was not used to generate study data, modify source conversations, or make clinical determinations.

The classification prompt instructed the model to rate each conversation on four prespecified domains using 0-2 scores and to apply explicit inclusion and exclusion criteria for mental health-related content. Prompts were developed iteratively before the final analysis using pilot examples, then fixed before classification of the validation sample and full corpus. The final prompt sequence, scoring rubric, and decision rules are provided below in Section 2 of the eMethods.

To evaluate classifier performance, two human raters independently coded a reference-standard sample of 370 conversations using the same conceptual framework. Human reviewers were trained on the scoring rubric and independently applied the finalized criteria to the validation sample. For conversations with discordant ratings, a third reviewer examined the conversation and the prespecified inclusion/exclusion criteria, and assigned the final adjudicated label. The adjudicated human label served as the reference standard for estimating classifier performance.

We calculated raw agreement and Cohen κ for human ratings, and estimated classifier sensitivity and specificity against the adjudicated human reference standard. To reduce AI-related methodologic bias and inaccuracy, we used prespecified definitions, human validation, adjudication of discordant human ratings, and transparent reporting of classifier performance. No identifiable patient information was included in the manuscript, tables, figures, or Supplement. The Harvard University institutional review board deemed the study exempt because the data were publicly available and deidentified.

2. **LLM Classifier Script**

Decide whether an AI-human conversation is Mental-Health Related (YES) or Not Mental-Health Related (NO), aligned to human expert judgments. Use the typology, rules, and examples below to prioritize precision (reduce false positives) while preserving sensitivity.

You must:

1. Apply the decision procedure.
2. Provide a brief rationale citing concrete spans from the conversation.
3. Assign YES or NO.
4. If YES, assign one or more categories from the typology.
5. Populate the output schema exactly.

## Core Principle

© 2026 McBain RK et al. *JAMA Network Open*.

A conversation is *Mental-Health Related* only if the user's intention is to seek or provide mental-health support, understanding, coping, risk/safety, or treatment navigation for themselves or for a real other person.

Mental-health *content* alone (e.g., academic, fictional, professional, or creative tasks) does not make the conversation mental-health related. The classifier must evaluate what the user is trying to accomplish, not simply what topics appear.

If the user is not seeking help for mental health concerns (their own or someone else's), the correct label is NO—even if the conversation contains mental-health terminology.

Intention Gate (Stage 1)

Before applying any category or score, determine whether the user is seeking mental-health support.

A. Classify as NO immediately if the user's intention is any of the following:

1. Content Creation or Writing Tasks
   Includes: essays, letters, poems, speeches, videos, stories, scripts, roleplays, fictional scenarios, dialogues, practice interviews, character sketches, personal statements, thank-you notes, school assignments.
   Example: "Write a thank-you note to my therapist." → NO.
   Example: "Generate a YouTube video script on coping with burnout." → NO.
2. Professional / Training / Educational Tasks
   Includes: clinical training, exam prep, OSCE, treatment-planning exercises, counselor scripts, supervision tasks, principal/teacher protocols, hypothetical case studies.
   Example: "In this medical scenario, which communication option is correct?" → NO.
3. System Design / Product Design / Tech / ML Tasks
   Includes: building chatbots, designing recommender systems, evaluating algorithms, writing specs, safety guidelines, knowledge graphs, user journeys.
   Example: "Help me design an AI to match people with therapists." → NO.
4. Administrative or Logistical Work not about *one's own* MH Care
   Includes: insurance forms, records summarization, progress note formatting, documentation writing, unless the user is doing it for their own or another real person's MH care.
   Example: "Reformat this psychiatry progress note." → NO.
5. Curiosity, Definitions, or Label-Seeking without Help Intent
   Example: "Is there a name for someone who acts childish?" → NO (unless seeking support).
6. Fictional or Hypothetical Emotional Content
   Any emotional distress that exists inside the text being generated rather than the user's lived context → NO.

B. Only proceed to "YES category assignment" if BOTH are true:

1. The user expresses or implies a real person's emotional, psychological, or behavioral difficulty.
2. The user is seeking support, guidance, coping, risk assessment, navigation, or understanding for that real person.

© 2026 McBain RK et al. *JAMA Network Open*.

If both conditions hold → continue to typology.

If not → Label NO, category = "None".

## Typology (titles, definitions, and mini-examples)

YES cases, should encompass one of the following categories below. If the conversation does not correspond to one of the categories below, it should be labeled NO. If the conversation is assigned YES by you, you must clearly assign one or more categories:

## 1. Crisis & Self-Harm Risk

Definition: Conversations centered on imminent or serious risk of self-harm or suicide—including clear statements of wanting to die, harm oneself, or active safety planning (e.g., removing means, contacting hotlines). Emotional urgency and a request for help or safety guidance must be present. Figurative uses ("that exam killed me") or general sadness without self-harm intent do not qualify.

Counts:
- "I found my old antidepressants and keep thinking about taking them all. I'm scared I'll actually do it tonight." → Expresses plan, timing, and fear; direct risk disclosure.
- "I threw out my blades but still want to cut—can you help me get through tonight?" → Active urge plus help-seeking for safety.

Doesn't Count:
- "That commute is killing me; I need coffee." → Pure idiom; no risk.
- "I'm sad and exhausted lately but not thinking of hurting myself." → Low mood, but explicitly denies self-harm.

## 2. Acute Distress & Help-Seeking (Non-Imminent)

Definition: Conversations where the user reports significant emotional distress (e.g., panic, overwhelm, intrusive worry, insomnia) and asks for concrete coping or guidance, but without imminent self-harm risk. To count, there must be a specific help-seeking intent (skills, routines, scripts, short-term plans). Mere venting ("stressed," "panicked") or mood color added to non-MH tasks does not qualify.

Counts:
- "Before presentations I shake and can't breathe. Can you walk me through a 2-minute grounding routine I can use in the waiting room?" → Distress + explicit request for a coping protocol.
- "I spiral at night and can't sleep. Please design a one-week 'lights-out' checklist to stop doom-scrolling." → Symptom focus + concrete behavior plan.

Doesn't Count:
- "Super stressed this week—anyway, which laptop should I buy?" → Distress is incidental; focus is shopping.
- "I panicked when the website crashed; how do I restore the database from backup?" → "Panic" is idiomatic; thread is pure tech support.

© 2026 McBain RK et al. *JAMA Network Open*.

## 3. Mood & Anxiety Symptoms as the Focus

Definition: Conversations where depressive or anxiety symptoms themselves are the subject—e.g., anhedonia, low mood, guilt, sleep/appetite change, excessive worry, rumination, panic-like somatic signs—and the user seeks understanding, assessment context, or symptom-management strategies. To count, symptoms must be central with a request for interpretation or coping (e.g., severity checks, monitoring plans, evidence-based skills). Mentions of "depressed/anxious" as idiom, or as brief color while pursuing unrelated logistics, do not qualify.

Counts:
- "For two months I've had no interest in hobbies, early-morning waking, and guilt about falling behind. Does this sound like depression, and what should I track before seeing a clinician?" → Symptom cluster + interpretation/next steps.
- "I worry about layoffs most days—racing heart, restlessness. Can we set up a 10-minute 'worry time' routine and a way to track triggers?" → Anxiety symptoms + concrete management plan.

Doesn't Count:
- "That final was depressing. Anyway, which streaming service is best?" → Idiomatic "depressing"; focus is media shopping.
- "I'm anxious about my trip; can you build the itinerary and book flights?" → Logistics dominate; no MH interpretation or coping ask.

## 4. Trauma, Grief, and Loss

Definition: Conversations focused on processing or coping with trauma or loss—including the emotional, cognitive, and behavioral consequences of events such as accidents, violence, bereavement, or other psychologically distressing experiences. The user typically seeks understanding, validation, or strategies to manage intrusive memories, grief, or emotional numbness. Merely mentioning that something sad happened, or discussing logistics after a death, does not qualify unless the focus shifts to emotional or mental recovery.

Counts:
- "Ever since the car crash, I can't sleep because I keep seeing it happen again. How can I stop the flashbacks?" → Focus on post-traumatic symptoms and coping.
- "My dad passed away last month, and I feel empty even though everyone says it'll get easier. Is that normal?" → Seeks understanding and emotional processing of grief.

Doesn't Count:
- "My grandmother died, and I need help filling out life insurance paperwork." → Focus is administrative, not mental health.
- "That news story about the earthquake victims made me so sad." → Expresses empathy, not personal trauma or grief coping.

## 5. Psychosis & Severe Mental Illness

Definition: Conversations focused on severe psychiatric symptoms such as hallucinations, delusions, disorganized thinking, mania, or other indicators of psychosis or bipolar disorder. The

© 2026 McBain RK et al. *JAMA Network Open*.

user typically seeks understanding, reassurance, or treatment guidance, often describing perceptual disturbances, paranoia, or extreme mood elevation. To qualify, the symptoms must be personal, persistent, and distressing. Casual or figurative uses of diagnostic terms ("that idea is schizophrenic," "I'm manic about this project") do not count.

Counts:
- "I hear a voice that comments on what I do. It's terrifying, and I don't know if I should tell my psychiatrist." → Describes active hallucinations and help-seeking about care.
- "I haven't slept in three nights, and I feel unstoppable and keep spending money I don't have. Could this be mania?" → Concrete symptom cluster + diagnostic inquiry.

Doesn't Count:
- "The movie plot was totally schizophrenic—it made no sense." → Idiomatic; not a mental health disclosure.
- "I'm bipolar about pizza—sometimes I love it, sometimes I hate it." → Joke; no clinical content.

## 6. Substance Use & Addiction

Definition: Conversations focused on alcohol or drug use (prescription or recreational) that involve cravings, relapse, withdrawal, or efforts to quit or moderate. The user may discuss detox, sobriety maintenance, or fear of dependence. To qualify, the focus must be behavioral health—how substance use affects mood, control, or functioning. Mentions of substances in casual, social, or informational contexts do not count.

Counts:
- "I'm three days sober and craving alcohol so badly tonight. What can I do to ride out the urge?" → Describes withdrawal and seeks coping support.
- "I've been taking oxycodone after surgery for weeks and I'm worried I'm dependent. How should I talk to my doctor?" → Substance concern + treatment navigation.

Doesn't Count:
- "Which wine pairs best with salmon?" → Culinary context only.
- "Is CBD legal to fly with?" → Legal/logistical focus; not about use or recovery.

## 7. Treatment Navigation: Therapy / Medications / Diagnosis

Definition: Conversations about accessing or managing mental health care, including therapy, psychiatric medications, diagnosis clarification, or insurance coverage for mental health services. The user seeks specific guidance on how to start, continue, or troubleshoot treatment. References to "therapy" or "meds" as slang or lifestyle metaphors do not count.

Counts:
- "I've been on sertraline for two weeks and still feel nauseous—should I call my doctor or wait it out?" → Medication management + care decision.
- "Can you help me find a CBT therapist in-network and suggest what to say in my first session?" → Therapy access + preparation steps.

© 2026 McBain RK et al. *JAMA Network Open*.

Doesn't Count:
• "Cleaning my kitchen is my therapy—what's the best mop?" → Metaphor; not clinical treatment.
• "My friend said I need meds for how messy my desk is!" → Joke; no genuine treatment discussion.

## 8. Neurodiversity & Developmental (ADHD / Autism / Related Conditions)

Definition: Conversations centered on neurodevelopmental or attentional conditions—such as ADHD or autism—where the user seeks understanding, assessment, medication guidance, or strategies for daily functioning. The focus is on managing cognitive, sensory, or organizational challenges, or navigating formal evaluation and accommodations. Mentions of these terms as humor, identity shorthand, or exaggeration do not qualify.

Counts:
• "I keep missing deadlines because I lose track of time when I'm hyper-focused. Can you help me design a reminder system that actually interrupts me?" → ADHD-related executive-function problem + skills request.
• "I think I might be autistic—loud spaces make me panic and I avoid eye contact. How can I ask for a workplace assessment?" → Self-assessment + help-seeking.

Doesn't Count:
• "My ADHD made me buy three more mugs—send help!" → Humor; not a treatment or coping discussion.
• "Autism memes are hilarious—have you seen this one?" → Social chatter; no mental health focus.

## 9. Psychosocial Stressors Impacting Mental Health

Definition: Conversations about external stressors (e.g., work burnout, relationship conflict, academic pressure, financial strain) that explicitly link these experiences to emotional or mental well-being. The user asks for coping strategies, boundaries, or perspective shifts. If the stressor is discussed purely for logistics (scheduling, travel, budgeting) with no mental health component, it does not count.

Counts:
• "My manager keeps messaging me late at night and I can't switch off. How do I set boundaries without risking my job?" → Work stress directly connected to emotional exhaustion and coping.
• "Since the breakup I can't eat or focus. Can you help me build a simple daily routine to start feeling normal again?" → Relationship loss framed through mood and recovery.

Doesn't Count:
• "My boss is impossible—rewrite my email so it sounds professional." → Communication task only.
• "We're moving next week; can you find a truck rental?" → Life logistics; no mental health component.

## 10. Mental Well-Being & Coping Skills (Non-Clinical)

© 2026 McBain RK et al. *JAMA Network Open*.

Definition: Conversations focused on building everyday emotional resilience—such as mindfulness, journaling, gratitude, stress reduction, or self-care routines—without necessarily referencing a diagnosis. The user seeks practical, skill-based support for emotional regulation or balance. If mindfulness or self-care terms are used mainly for shopping, aesthetics, or travel planning, it does not count.

Counts:
- "I get tense after long meetings—can you design a 10-minute reset routine I can use before starting my next task?" → Focus on coping and emotional regulation.
- "I'd like to start journaling to calm my thoughts. How can I make it a daily habit without it feeling forced?" → Intentional well-being plan.

Doesn't Count:
- "I'm looking for a pretty mindfulness journal—what brand has the best paper?" → Shopping focus.
- "Is there a discount for the Calm app?" → Commercial interest, not coping.

## 11. Third-Party Concern (Supporting Others)

Definition: Conversations where the speaker seeks help supporting another person's mental health—for example, a friend, partner, or child—through recognition, communication, or referral. The goal is guidance on what to say or do for someone else's well-being. Routine caregiving, gift planning, or logistics without emotional or safety focus do not count.

Counts:
- "My roommate hasn't been eating or leaving her room. How can I check in without making her defensive?" → Third-party distress + supportive communication plan.
- "My teenager said she doesn't care if she wakes up tomorrow. What should I do right now?" → Concern for safety + crisis guidance.

Doesn't Count:
- "My partner is stressed—what's a good anniversary restaurant?" → Gift planning, not support for mental health.
- "My friend just got laid off—help me write a LinkedIn recommendation." → Career support only.

## 12. Physical–Mental Comorbidity & Body-Image / Eating

Definition: Conversations linking physical health or body-image issues to emotional well-being, such as chronic pain, fatigue, eating behaviors, or sleep disturbance. The focus is on how physical conditions affect mood, coping, or self-esteem, or how to manage body-related anxiety. Purely medical, nutritional, or fitness-mechanical questions do not qualify.

Counts:
- "Since my surgery, constant pain makes me snap at people and I hate myself for it. How can I manage my mood when I'm hurting?" → Connects physical symptoms with emotional strain and coping.
- "I keep restricting after workouts because I'm afraid of gaining weight. How do I stop the binge-restrict cycle?" → Body-image concern + help-seeking.

© 2026 McBain RK et al. *JAMA Network Open*.

Doesn’t Count:
- “What’s the safest dose of ibuprofen after wisdom-tooth extraction?” → Medical guidance only.
- “How many calories are in a protein shake?” → Nutritional info; no mental health link.

## 14. Mental-Health Admin / Logistics

Definition: Conversations addressing administrative tasks directly tied to mental-health care—such as finding in-network therapists, understanding CPT/ICD codes, submitting superbills, or verifying insurance coverage for counseling or psychiatry. Routine paperwork, finance, or travel topics unrelated to mental-health services do not count.

Counts:
- “How do I file an out-of-network claim for my therapy sessions using CPT 90834?” → Administrative support for mental-health care.
- “Can you check if teletherapy is covered before my deductible on my PPO plan?” → Insurance navigation for therapy.

Doesn’t Count:
- “I’m stressed about my taxes—how do I fill out Schedule C?” → General admin; not mental-health care.
- “Can you renew my passport faster if I say it’s urgent?” → Travel paperwork; irrelevant to mental health.

## Calibration Snippets (hard negatives to reduce False Positives)
Label NO for each unless there is a clear MH pivot:
- “This deadline is killing me. Anyway, can you fix my Python error?”
- “Had a panic when the website crashed—what’s the rollback command?”
- “That film was depressing. Which streaming plan is cheaper?”
- “I need the therapy of reorganizing cables—best Velcro ties?”
- “Breakdown of costs by quarter, please.”
- “My ADHD made me buy another mug—any discount code?”

## Tie-Breaker Rules

1. If the user is creating content for an audience, the default is NO, even if the content is about mental health.
2. If the user is acting in a professional or academic role, default to NO unless they are seeking help for a real individual’s mental health (e.g., “a student said X—what should I do now?” → YES).
3. If distress/diagnostic terms appear in fictional text, or in an example the user wants to generate, default to NO.
4. If mental-health terminology is used for classification, coding, analytics, or ML design, default to NO.
5. To count as YES, the user's intent must include actionable help-seeking or care-seeking for a real person.

## Examples

© 2026 McBain RK et al. *JAMA Network Open*.

Example A (NO: Content creation)
User: “Write a heartfelt letter from someone who feels anxious and depressed, asking for guidance.”
→ NO (fiction).
Example B (NO: Professional scenario)
User: “On this psychiatry OSCE question, what should the clinician say to a suicidal patient?”
→ NO (exam prep).
Example C (YES: Third-party real person)
User: “My teenage son said he doesn’t care if he wakes up tomorrow. What do I do right now?”
→ YES → thirdparty.
Example D (YES: Personal mental health)
User: “I’ve been having panic attacks at night—how can I calm down enough to sleep?”
→ YES → distress.
Example E (NO: System design)
User: “Help me build a chatbot that can identify suicidal language.”
→ NO.
Example F (YES: Treatment navigation)
User: “I started sertraline last week and feel nauseous. Should I call the doctor?”
→ YES → treatment.

## Scoring Domains

**topicality (T)**
- 0 = Not about mental health, no clinical/problem context
- 1 = Mental health or emotional wellness mentioned in passing, not central topic
- 2 = Mental health (disorder, coping, support, treatment) is central focus

**intent (I)**
- 0 = No help-seeking/giving behavior related to mental health
- 1 = Discussion of general well-being or minor emotional issues (without disorder/coping/treatment)
- 2 = Explicitly seeking/giving mental health advice, treatment, or crisis support

**clinicality (C)**
- 0 = No clinical terms/diagnosis/therapy/risk discussion
- 1 = Occasional use of terms like “depressed,” “anxious,” “stressed” but not clinical or problem-focused
- 2 = Specific clinical terms, diagnoses, treatments (CBT, SSRIs), or professional care

**affect_risk (R)**
- 0 = No genuine distress/impairment, only everyday emotions (love, happiness, ordinary sadness)
- 1 = Noticeable emotional distress/impairment in context of mental health
- 2 = Severe distress, crisis, suicide, self-harm, or explicit risk

**user_role**
- "patient" = Individual discussing own mental health problem/concern/diagnosis/treatment/coping

© 2026 McBain RK et al. *JAMA Network Open*.

- "medical_professional" = Clinician/therapist giving professional care/advice
- "peer_supporter" = Non-professional offering support specific to mental health distress
- "other" = Creative/fictitious scenario, ordinary relationship/love/storytelling, not mental health related

**is_english**
- YES: entire conversation is in english
- NO: conversation contains non-English

## Output Format

If you believe the conversation is mental health related, including passage of the intentionality criteria, assign one of the categories described above. Here are the shorthand bins: "crisis" | "distress" | "mood" | "trauma" | "psychosis" | "substance" | "neurodiversity" | "psychosocial" | "mental" | "thirdparty" | "physical" | "meta" | "logistics". Assign as "None" otherwise.

Respond ONLY with valid JSON:

```json
{
  "is_english": boolean,
  "user_role": "patient" | "medical_professional" | "peer_supporter" | "other",
  "scores": {
    "topicality": integer (0-2),
    "intent": integer (0-2),
    "clinicality": integer (0-2),
    "affect_risk": integer (0-2)
  },
  "reasoning": "1-2 sentence explanation of classification decision in accordance to the framework",
  "category": "crisis" | "distress" | "mood" | "trauma" | "psychosis" | "substance" | "treatment" | "neurodiversity" | "psychosocial" | "mental" | "thirdparty" | "physical" | "meta" | "logistics" | "None",
  "is_mental_health_related": boolean,
}
"""
```

© 2026 McBain RK et al. *JAMA Network Open*.
