# Supplementary material for: Prevalence of Mental Health Discussions in Publicly Available Generative AI Conversations
Source: JAMA Netw Open. 2026 Aug 24;9(8):e2630635. doi: 10.1001/jamanetworkopen.2026.30635 (PMC13504430; doi:10.1001/jamanetworkopen.2026.30635)
Supplement: Supplementary file 2 [file jamanetwopen-e2630635-s002.pdf]

Data Sharing Statement

McBain. Prevalence of Mental Health Discussions in Publicly Available Generative AI Conversations. *JAMA Netw Open*. Published August 24, 2026.
doi:10.1001/jamanetworkopen.2026.30635

Data
Data available: No

Additional Information
Explanation for why data not available: The data are available on a public database, as noted by the authors in the methods section. The classifier script is also available as an appendix associated with this article.
